# Nrf2 activation as target to implement therapeutic treatments

**DOI:** 10.3389/fchem.2015.00004

**Published:** 2015-02-02

**Authors:** Velio Bocci, Giuseppe Valacchi

**Affiliations:** ^1^Department of Biotechnologies, Chemistry and Pharmacy, University of SienaSiena, Italy; ^2^Department of Life Sciences and Biotechnology, University of FerraraFerrara, Italy

**Keywords:** oxidative stress, antioxidants, pathologies, calorie restriction, ozone

## Abstract

A chronic increase of oxidative stress is typical of serious pathologies such as myocardial infarction, stroke, chronic limb ischemia, chronic obstructive pulmonary disease (COPD), type II-diabetes, age-related macular degeneration leads to an epic increase of morbidity and mortality in all countries of the world. The initial inflammation followed by an excessive release of reactive oxygen species (ROS) implies a diffused cellular injury that needs to be corrected by an inducible expression of the innate detoxifying and antioxidant system. The transcription factor Nrf2, when properly activated, is able to restore a redox homeostasis and possibly improve human health.

## Introduction

Today a considerable fraction of the human population undergoes excessive feeding while the remaining is either undernourished or starving. Cardiovascular diseases (Di Paolo et al., 2005), COPD (Borrelli and Bocci, 2014) type II-diabetes (Feldman, 2003), smoking, drug addiction and reduced physical activity are situations widely diffused: the inexorable progression of these diseases is due to an enhancement of dysmetabolic processes such as the activation of NADPH-oxidases, xantine oxidase and Toll-like receptors (Gill et al., 2010) characterized by an excessive release of both ROS and nitrogen oxidative species (NOS), such as O^−^_2_, H_2_O_2_, OH^−^, ONOO^−^ which, subjected to Michael addition reactions with lysine, histidine and cysteine residues lead to protein carbonylation (Levine, 2002). Moreover hyperglycemia allows the reaction of the carbonyl group of reducing sugars to react with free amino groups of hemoglobin to form Schiff-base intermediates, which undergo the Amadori rearrangements to damageous ketoamine derivatives. Their degradation into glyoxal and methylglyoxal allows the formation of inter-intra-molecular cross-links with long lived proteins to form the AGES. These compounds, deposited in the arterial wall and nerve proteins, switch on an oxidative stress establishing a vicious inflammatory loops. These processes lead to insulin resistance in type II-diabetes, macular and lens degeneration (Li et al., 2009), immune dysfunction, impaired cell respiration associated to a relevant decline of antioxidant defenses (Valko et al., 2007). The metabolic deregulation leads to a further decline of antioxidant proteins and to a number of metabolic diseases. In the past it was thought that the administration of several antioxidants may reduce the damage but this is a passive therapy accompanied by a scarce bioavailability or inability to reach the appropriate target or to stimulate the innate antioxidant response (Bocci and Valacchi, 2013). Moreover cytokines such as IL-1, IL-6, IFN gamma, TNF alpha, via their binding to membrane cell receptors, further trigger ROS production and this situation has been well documented in ischemia and reperfusion injury, atherosclerosis, rheumatoid arthritis, pulmonary and neurodegenerative diseases and type II-diabetes responsible of the death of many millions people.

## How a chronic oxidative stress can be corrected?

It is now well established that one theory that related the development of pathologies (mainly during aging) concerns the progressive cellular loss of functions. One burning question regards the variability (heterogeneity) of humans to develop diseases, and the answer can be found in the individual different genetic interaction, environments and life events that shape our body to certain physiological responses.

Nowadays, the hormesis concept has been revaluated and stress-induced hormesis may be one effective way to prevent cell damage and promoting healthy aging. Basically, the term hormesis describes a process that results in ameliorating and improve cellular stress resistance, survival, and longevity in response to sub-lethal levels of stress. It has been proposed that hormesis can promote healthy aging. Overall a similar concept described for the activation of Heat shock Proteins (HSPs) which are as part of the cell's internal repair mechanism. Indeed HSPs are also called “stress-proteins” and respond to several stress not only related to temperature variation. heat, cold, and oxygen deprivation by activating several cascade pathways.

Several hormetic stressors have been describe, including, but not limited to moderate exercise, calorie restriction, pro-oxidant exposure.

During the last three decades the positive effect of practicing moderate physical exercise has been deeply studied, confirming its possible beneficial effect. This concept is based on the fact that performing exercise requires an increase in ATP production with the subsequent increase in metabolism that leads to augment the cellular ROS production. This phenomena will be able to activate the cellular defensive mechanisms, in response to increased ROS levels. This is confirmed by the fact that mild oxidative stress generated through regular exercise has been associated with a decreased incidence of ROS-related diseases, such as CDV and type II diabetes.

The beneficial effect of moderate exercise has become popular and provided that a moderate exercise, possibly accompanied by a moderate calorie restriction is performed daily, appears valid (Kang et al., 2009; Little et al., 2010). While a very intensive exercise may be detrimental, a moderate exercise induces mitochondrial biogenesis and an increased synthesis of super-oxide dismutase (SOD) and heme-oxygenase-1 (HO-1).

In fact, calorie restriction has an apposite effect, it is able to slow the metabolism and to reduce ROS production and it has been strongly inversely correlated to the development of ROS-related disease. The first studies using laboratory mice kept at a restricted caloric intake allowed a significant reduction of age-related diseases and prolonged life. Several studies by Fontana et al. (2004), Fontana (2008, 2009) in humans showed that long term calorie restriction is very protective and reduces atherosclerosis and mortality. Nutrient deprivation appears to activate SIRT 1 (Cohen et al., 2004), SIRT 3 (Sack, 2012) and SIRT 6 (Kanfi et al., 2008, 2012) and promotes a longer survival. The usual calorie intake of 2200–2400 calories/die must be constantly reduced to about half value and provided that the diet is equilibrated, it may be accepted by a number of people.

Also diet manipulation has been correlated to decrease the development of several pathologies. Indeed, administration of selected chemical compounds may reduce the chronic oxidative stress.

Recently it has appeared interesting to evaluate the value of oral curcumin in a dosage of 500 mg/day, which, in conjunction with a daily exercise and dietary reduction, displays anti-inflammatory and anti-oxidant activity (Gupta et al., 2012). Unfortunately curcumin has a low bioavailability and remain uncertain if it is able to reverse the pathology of chronic diseases. Resveratrol may modulate lifespan and metabolic disorders (Baur and Sinclaire, 2006). L-sulforaphane (SFN) is an isothiocyanate found in broccoli able to induce phase II detoxification enzymes and the synthesis of GSH but once again the dosage able to reverse a chronic diseases remains uncertain (Finley et al., 2005). There are also isoflavones, epigallocatechin-3-gallate (EGCG) present in green tea and finally a dosage of 600–1800 mg of N-acetylcysteine appears useful in pulmonary diseases.

Recently the study of therapeutic gasses such as NO (Knowles and Moncada, 1992), H_2_S (Calvert et al., 2009), CO (Pannen et al., 1998; Nakao et al., 2009), and Hydrogen (Ohsawa et al., 2007) have become relevant in selected pathologies.

During the last two decades several approaches have been suggested and although the evidences of their beneficial effect are not yet completely clear, the implementation of these approaches can be the way to ameliorate their effects on human health.

Besides the above mentioned approaches, other approaches seems to be involved in the activation of cellular hormetic process.

Hyperbaric oxygen (HBO) therapy has recently been promoted as an approach to slow aging. HBO treatment consists in breathing 100% oxygen under increased atmospheric pressure. In a HBO therapy chamber, the air pressure is increased to three times higher than normal air pressure. Under these conditions, the lungs can gather more oxygen than would be possible breathing pure oxygen at normal air pressure. Although the hemoglobin is saturated, being the blood hyperoxygenated can dissolve oxygen within the plasma throughout the body. Therefore, the therapeutic principle behind HBO stems from increasing the partial pressure of oxygen in the tissues of the body.

Currently it is used to treat CO poisoning, delayed radiation injuries, decompression sickness, as well as non-healing diabetic wounds and others. Furthermore, HBO preconditioning has been shown to have hormetic effects on stress resistance and can increase the longevity of *C. elegans*. Although the mechanism through which HBO increases longevity is unclear, it is likely to be related to the fact that HBO can induce low levels of ROS which are able to induce “mild stress” able itself to trig the transcription of protective genes.

Similar concept can be applied to the use of ozonetherapy. Hans Wolff (1927–1980) was the first physician to develop ozonated autohemotherapy (AHT) by insufflating *ex vivo* a gas mixture (O_2_:95%–O_3_:5%) into a volume of about 100 ml of blood withdrawn from a patient. Our scientific studies (Bocci, 2006; Bocci and Aldinucci, 2006; Travagli et al., 2007; Bocci et al., 2009a,b, 2010, 2011a,b; Sagai and Bocci, 2011) have been useful to limit the ozone dosage and to fully understand the biological mechanisms of action. As ozone is extremely reactive, it must be used in micrograms. The right concept is that a small acute oxidative stress will induce a positive antioxidant response by the patient. Indeed, pharmacologically it acts in a hormetic fashion (Bocci et al., 2011b; Calabrese, 2013) according an inverted V shape curve. A lower dosage would act as a placebo because human plasma contains several antioxidants able to neutralize ozone. The lipoperoxidation of unsaturated fatty acids (PUFA) with ozone, present in the plasma leads to the formation of aldehydes and H_2_O_2_ (Pryor et al., 1995), which represent the critical messengers of ozone. Thus ozone disappears from the plasma within 1–2 min. H_2_O_2_ may reach a concentration of a few micrograms in plasma because it freely enters into all the blood cells where activates several biochemical pathways before being totally reduced to H_2_O by catalase, thioredoxin and GSH (Antunes and Cadenas, 2000). H_2_O_2_ significantly activates both glycolysis and glycerophosphomutase in erythrocytes thus increasing the level of 2,3 diphosphoglycerate (2,3-DPG). This is an important effect because slowly the oxyhemoglobin curve will be shifted to the right with the advantage of improving the release of oxygen into any ischemic tissue (Bocci et al., 2009a). Moreover, leukocytes increase phagocytosis and platelets may release some growth factors useful in ischemic diseases (Valacchi and Bocci, 1999, 2000).

During the infusion of the ozonated blood the activation of NO synthase will also expand the oxygenation of ischemic tissues (Valacchi and Bocci, 2000). In the plasma oxidated antioxidants such as uric acid will be eliminated as allantoin while ascorbic acid, thioredoxin and GSH will soon be reduced (Mendiratta et al., 1998) thus rapidly restoring the potent antioxidant system of plasma.

The dogma that ozone should not be used in medicine is however untenable because human blood, which has a wealth of antioxidants (about 1.3–1.8 mM) is never damaged by the minimal ozone therapeutic concentrations (Bocci et al., 2009a; Bocci and Valacchi, 2013).

During the infusion of the ozonated blood, the second stress phase takes place because the hydroperoxides are further broken down by GSH transferases or lead to the formation of alkenals such as 4-HNE (from N6 PUFA) or HHE (from N3 PUFA) (Long and Picklo, 2010). These alkenals are electrophilic, amphipathic molecules which forms adducts with cysteine, GSH or with Cys 34 present in the domain 1 of albumin. Moreover alkenals are metabolized by GSH-transferase, aldehyde dehydrogenase (Awasthi et al., 2004), and are also excreted via bile (Laurent et al., 1999) and renal excretion (Alary et al., 1995). The remaining submicromolar concentration of alkenals, bound to the cysteineof albumin, becomes the second very important messenger as the adduct will be released in variousorgans of the body and represents the signal for a variety of cells of a transitory, acute oxidative stress.

## Is the Keap-Nrf2 pathway able to store a normal redox system?

Usually evolution has progressed with the production of oxidative insults and, whenever possible, the organization of an appropriate defense system. Unfortunately, during the mentioned chronic diseases, orthodox therapy, although effective, is only partially able to reduce the progressive damage due to the chronic oxidative stress.

Moi et al. (1994) discovered a new transcription factor denominated Nuclear factor erythroid 2-related (Nrf2) which is a protein with a molecular weight of 95–110 kilodalton (Lau et al., 2013) belonging to the basic leucine zipper transcription factor.

Under normal conditions it is repressed by a protein with 624 amino acids called Keap1 (Kelch-like erythoid cell derived protein) containing as many as 25 cysteines, which is an adaptor protein for a Cullin 3 (Cul3)-dependent ubiquitination and degradation of Nrf2. Normally the complex Nrf2-Keap1 is present in the cytoplasm with a half-life of about 20 min. because, if it is not used, is degraded by the proteasome. Further on this complex has been examined by several authors (Talalay et al., 1995; Itoh et al., 1997; Motohashi and Yamamoto, 2004; Jung and Kwak, 2010) and recently the critical role of Nrf2 has become evident. Suzuki et al. (2013) have clarified the role of Cys151, Cys 273, Cys 288, Cys 226, Cys 613, and of Cys 434. These cysteine residues are the sensor system and different electrophiles are able to recognize distinct cysteine residues thus making Keap1 a very sensitive sensor system. As an example SFN binds to Cys151, metals to Cys 613, 8-nitro-cGMP binds to Cys434 and alkenals select Cys 273 and Cys 288. The blocking of these cysteine causes the loss of repression of Nrf2 by Keap1 and therefore Nrf2 becomes free and moves into the nucleus where it dimerizes with members of the small Maf family and binds to the electrophil response elements (ARE) located in the regulatory regions of cellular defense enzyme genes. It has been shown that Nrf2 activation can be a common denominator among the above mentioned cellular hermetic activators. In fact the ability of exercise to induce ROS activates Nrf2, which increase the expression of antioxidant enzymes, such as GPx, GST, and HO-1 (de Lemos et al., 2012). In addition in an elegant study, Muthusamy et al. (2012) were able to show that acute exercise activates Nrf2 in the myocardial tissue and that disruption of Nrf2 (using KO animals) increases the susceptibility of myocardial tissue to OS-induced damage, suggesting that Nrf2 activation could be a potential therapeutic protective target. In addition, the ability of CR to attenuate vascular oxidative damage seems to be also linked to the activation of Nrf2/ARE pathway, which may serve as an endogenous vascular antioxidant system reinforcing the cellular oxidative stress tolerance (Ungvari et al., 2008).

In line with this, our recent work (Pecorelli et al., 2013) using endothelial cells has shown that H_2_O_2_, 4-HNE and ozonated human serum show a rapid Nrf2 activation. Other mechanisms such as the use of butein, the activation of the ERK pathway, NADPH oxidase 4, accumulation of fumarate causing Keap1 succination and also phosphorilation of Nrf2 are all able to free Nrf2 (Kensler et al., 2007; Jung and Kwak, 2010; Adam et al., 2011; Yang et al., 2011, 2012). Finally, the activation of Nrf2 by HBO therapy has also been also documented in several recent papers (Godman et al., 2010; Soejima et al., 2012; Xu et al., 2014), therefore on the whole it is clear that several low intensity or hormetic biological stressors can activate the Nrf2 system. The activation of Nrf2 induces an increased synthesis of a number of phase2-proteins and antioxidant enzymes able to counteract the chronic oxidative stress as follows:

1. Activation of the synthesis of antioxidants able to detoxify an excess of ROS such as catalase, superoxide-dismutase (SOD), GSH-peroxidases, GSH-reductase, GSH-transferase, NADPH-quinone oxidoreductase (NQO1), Cytochrome P450 monooxygenase system, thioredoxin and thioredoxin reductase and HSP70.
2. Activation of the GSH synthase allows a marked increase of the GSH intracellular level, which is very protective.
3. The enhancement of the synthesis and levels of phase II enzymes such as UDP-glucuronosyltransferase, N-acetyltransferases and sulfotransferases.
4. The upregulation of HO-1 which is a very protective enzyme with a possible increase of CO which in combination with NO allows vasodilation of ischemic tissues.
5. Reduction of iron overload via elevated ferritin and bilirubin as a lipophilic antioxidant. Both the II phase proteins and the antioxidants are capable to correct the chronic oxidative stress and to restore a normal redox system.

### Nrf2 paradox

Paradoxically, recent papers have suggested a dark side of Nrf2 (Wang et al., 2008) that consist in the ability of cancer cells to acquire a growth advantage thank to the loss of Keap1 leading therefore to Nrf2 activation. This was confirmed by the fact that several kind of cell cancer showed a clear Nrf2 up-regulation (skin, breast, lung, prostate etc…) (Zhang, 2010). Nrf2 overexpressed provides therefore a clear advantage to cancer cells making them less susceptible to OS damage and reinforcing their chemoresistance. For instance, several phase II enzymes under Nrf2 control have been overexpressed in cancer cells; HO-1 overexpression was present in prostate and brain cancer beside renal cancer (Goodman et al., 1997); NQO1 upregulation was detected in hepatoblastoma, colon cancer, breast cancer, and non-small cell lung cancer (Maines and Abrahamsson, 1996).

The reasons behind the overactivation of Nrf2 in cancer cells are not well clear, although several potential mechanisms have been proposed such as Keap1 mutation that will accumulate Nrf2 in the nucleus and preventing also its re-uptake (No et al., 2014).

Mutation of Keap1 leads to the overexpression of Nrf2 and its target genes. Mutation of Keap1 can result in accumulation of Nrf2 in nucleus through decreased degradation or inhibition of nuclear export (Gañán-Gómez et al., 2013). In addition mutation in genes involved in Nrf2 activation such as EGFR, Myc, etc… have been fund in cancer cells (Kim et al., 2010). Furthermore, epigenetic modification in both Keap1 and Nrf2 can also activate Nrf2 as demonstrated in malignant glioma, colon cancer, and breast cancer (Muscarella et al., 2011; Hanada et al., 2012; Barbano et al., 2013).

## Conclusions

**Figure 1:**
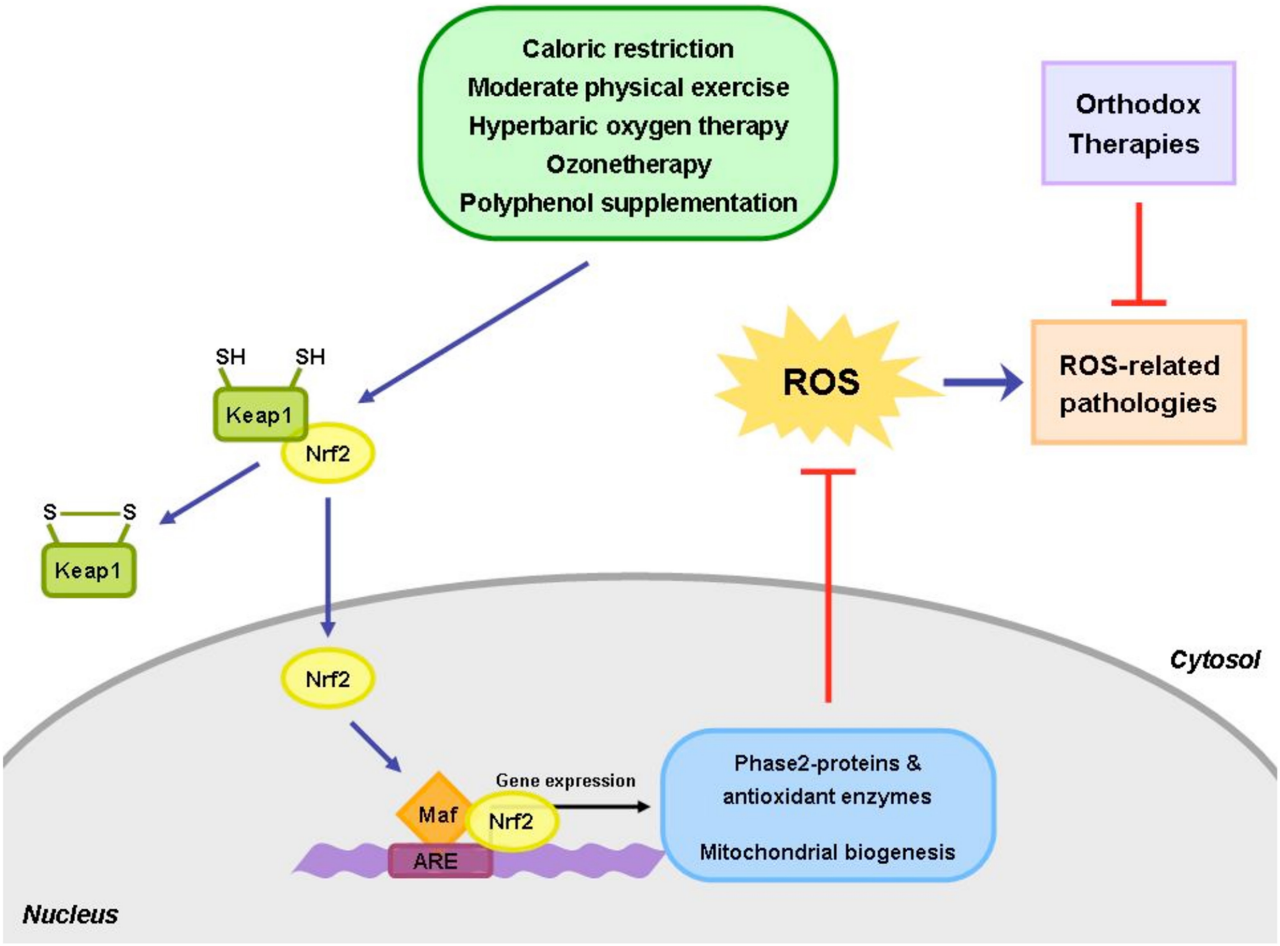
**The use of alternative approaches, such as caloric restriction, exercise and healthy diet, together with the conventional medical treatments, could be useful to help patients restore and maintain their healthy physiological state**. The homeostasis between the oxidant and antioxidant species is frequently perturbed in a number of human pathological conditions. Hence, the integrative approaches, able to re-establish the redox balance, could play a key role in restoring the cellular homeostasis and reduce the side effects of the orthodox medicine.

One of the main processes involved in diseases development and its progression is represented by the alteration of cellular redox homeostasis. The ability of an organism to reestablish the correct homeostasis and to faith the increased oxidative levels can be the critical way to survive and to avoid potential cellular damage. The activation of Nrf2 by several different mechanisms (calorie restriction, ozonetherapy, HBO, physical exercise) can be a way to improve life health (Figure 1). In addition, the implementation of the above mentioned strategies to the conventional therapies should be new approach to follow to cure several diseases. Of course we need to take into consideration that Nrf2 activation follows an hermetic rule. Constitutively expressed Nrf2 can promote cancer cell proliferation and protect cells against oxidative stress and therapeutic agents. It must be kept in mind that chemopreventive agents including various phytochemicals can induce chemoresistance and tumor progression by activating the Keap1-Nrf2 pathway.

### Conflict of interest statement

The authors declare that the research was conducted in the absence of any commercial or financial relationships that could be construed as a potential conflict of interest.
